# ROTS: An R package for reproducibility-optimized statistical testing

**DOI:** 10.1371/journal.pcbi.1005562

**Published:** 2017-05-25

**Authors:** Tomi Suomi, Fatemeh Seyednasrollah, Maria K. Jaakkola, Thomas Faux, Laura L. Elo

**Affiliations:** 1 Turku Centre for Biotechnology, University of Turku and Åbo Akademi University, Turku, Finland; 2 Department of Future Technologies, University of Turku, Turku, Finland; 3 Department of Mathematics and Statistics, University of Turku, Turku, Finland; Universite de Montreal, CANADA

## Abstract

Differential expression analysis is one of the most common types of analyses performed on various biological data (e.g. RNA-seq or mass spectrometry proteomics). It is the process that detects features, such as genes or proteins, showing statistically significant differences between the sample groups under comparison. A major challenge in the analysis is the choice of an appropriate test statistic, as different statistics have been shown to perform well in different datasets. To this end, the reproducibility-optimized test statistic (ROTS) adjusts a modified *t*-statistic according to the inherent properties of the data and provides a ranking of the features based on their statistical evidence for differential expression between two groups. ROTS has already been successfully applied in a range of different studies from transcriptomics to proteomics, showing competitive performance against other state-of-the-art methods. To promote its widespread use, we introduce here a Bioconductor R package for performing ROTS analysis conveniently on different types of omics data. To illustrate the benefits of ROTS in various applications, we present three case studies, involving proteomics and RNA-seq data from public repositories, including both bulk and single cell data. The package is freely available from Bioconductor (https://www.bioconductor.org/packages/ROTS).

“This is a *PLOS Computational Biology* Software paper.”

## Introduction

Differential expression analysis between two groups of samples is perhaps the most common type of analysis that is performed on various types of omics data. The aim of differential expression analysis is to detect features (e.g. genes or proteins) showing statistically significant changes between the groups. A commonly used approach has been the Student’s *t*-test, which has been later shown not to be the most optimal solution in many cases [1, 2]. Although a number of alternative test statistics have therefore been introduced [3–5], a major practical challenge remains that the different statistics perform well in different datasets [6–9] and there is no general agreement on how to make an appropriate choice of the statistic *a priori*.

To address this need, we have introduced a reproducibility-optimized test statistic (ROTS) that optimizes the choice of the statistic directly from the data instead of using a fixed predefined statistic [10]. This is done by maximizing the overlap of top-ranked features in group-preserving bootstrap datasets among a family of *t-type* statistics. In particular, ROTS optimizes the ranks of the features because usually the final ranking determines if the differentially expressed features are selected for further validation studies.

The ROTS method has already been used in various applications, such as microarrays [10], mass spectrometry proteomics [11] as well as bulk and single-cell RNA-seq [9, 12], and its competitive performance has been shown against other tools for differential expression analysis. Here we introduce a Bioconductor R package ROTS for performing differential expression analysis using the ROTS method and demonstrate the applicability of the method in three diverse case studies. The R package together with detailed documentation is freely available from Bioconductor.

## Design and implementation

### Algorithm

ROTS optimizes the reproducibility of top-ranked features in group-preserving bootstrap datasets among a family of modified *t*-statistics:

$$d_{\alpha }=\frac{\left|\bar{x_{1}}-\bar{x_{2}}\right|}{\alpha_{1}+\alpha_{2}s}$$ (1)

where $\left|\bar{x_{1}}-\bar{x_{2}}\right|$ is the absolute difference between the group averages, *α*_1_ and *α*_2_ are non-negative parameters to be optimized, and *s* is the pooled standard error [10]. Special cases of ROTS are the ordinary *t*-statistic (*α*_1_ = 0, *α*_2_ = 1) and the signal log-ratio (*α*_1_ = 1, *α*_2_ = 0). The optimal statistic is determined by maximizing the reproducibility Z-score:

$$Z_{k}\;\left(d_{\alpha }\right)=\frac{R_{k}\;\left(d_{\alpha }\right)-R_{k}^{0}\;\left(d_{\alpha }\right)}{s_{k}\;\left(d_{\alpha }\right)}$$ (2)

over a lattice *α*_1_ ∈ {0, 0.01, …, 5}, *α*_2_ ∈ {0, 1}, *k* ∈ {1, 2, …, *G*}. Here, *R*_*k*_ (*d*_*α*_) is the observed reproducibility of statistic *d*_*α*_ at top list size *k* in bootstrap datasets, $R_{k}^{0}\;\left(d_{\alpha }\right)$ is the corresponding null reproducibility in randomized datasets permuted over samples, *s*_*k*_ (*d*_*α*_) is the standard deviation of the bootstrap distribution, and *G* is the total number of features in the data. Reproducibility is defined as the average overlap of *k* top-ranked features over pairs of bootstrapped datasets.

The final ROTS output is calculated from the original data using the optimized parameters *α*_1_ and *α*_2_ giving the highest reproducibility Z-score. The false discovery rate (FDR) is estimated by randomly permuting the sample labels.

### Software features

The ROTS package is freely available from Bioconductor (https://www.bioconductor.org/packages/ROTS) and runs in R environment. Both the package and the R environment can be used on Windows, MacOS or UNIX platforms. After installing the package, the differential expression analysis can be performed within R. A preprocessed and appropriately normalized data matrix is required for input with columns representing different samples and rows representing the features. It can be various types of omics data, such as gene expression microarray data, RNA-seq data or mass spectrometry based proteomics data. As an example, an excerpt from a typical expression matrix of a label-free proteomics study is shown in Table 1, containing log scaled protein abundances of three replicates from two sample groups of the shotgun ‘profiling standard sample set’ [13].

**Table 1:** Example layout of expression data to be used as input for ROTS, where columns represent different samples and rows represent the features.

Feature	A1	A2	A3	B1	B2	B3
1	19.263	19.213	19.151	19.138	19.168	19.328
2	25.950	25.935	25.950	24.040	24.058	24.078
3	21.077	20.982	21.101	21.255	21.263	21.328
4	20.691	20.531	20.470	20.921	20.902	20.911

To perform differential expression analysis on an expression matrix (here data), only one line of code is required after loading the package:

library(ROTS)

rots.out <- ROTS(data, groups = c(0,0,0,1,1,1), B = 1000, K = 500)

Here the vector groups defines the columns of the data matrix belonging to the two different sample groups under comparison, B denotes the number of bootstraps to perform, and K is the maximum top list size to consider in reproducibility optimization. Setting this number to a smaller value may improve the running time drastically. However, we recommend that the value should always be considerably higher than the number of features expected to be differentially expressed.

The generated ROTS object (here rots.out) contains the test statistics and additional details for all the features in the input data, including the optimized parameters a1 and a2. If the reported top list size *k* (rots.out$k) is close to the given parameter K, it suggests that the maximum top list size to be tested might have been too small, and increasing it should be considered. The reproducibility value (rots.out$R) and the reproducibility Z-score (rots.out$Z) are also included. All the results including *p*-values (rots.out$pvalue), false discovery rates (rots.out$FDR) or fold changes (rots.out$logfc) can be exported by the user and used for further external analysis, such as gene-set or pathway enrichment analysis.

**Fig 1:**
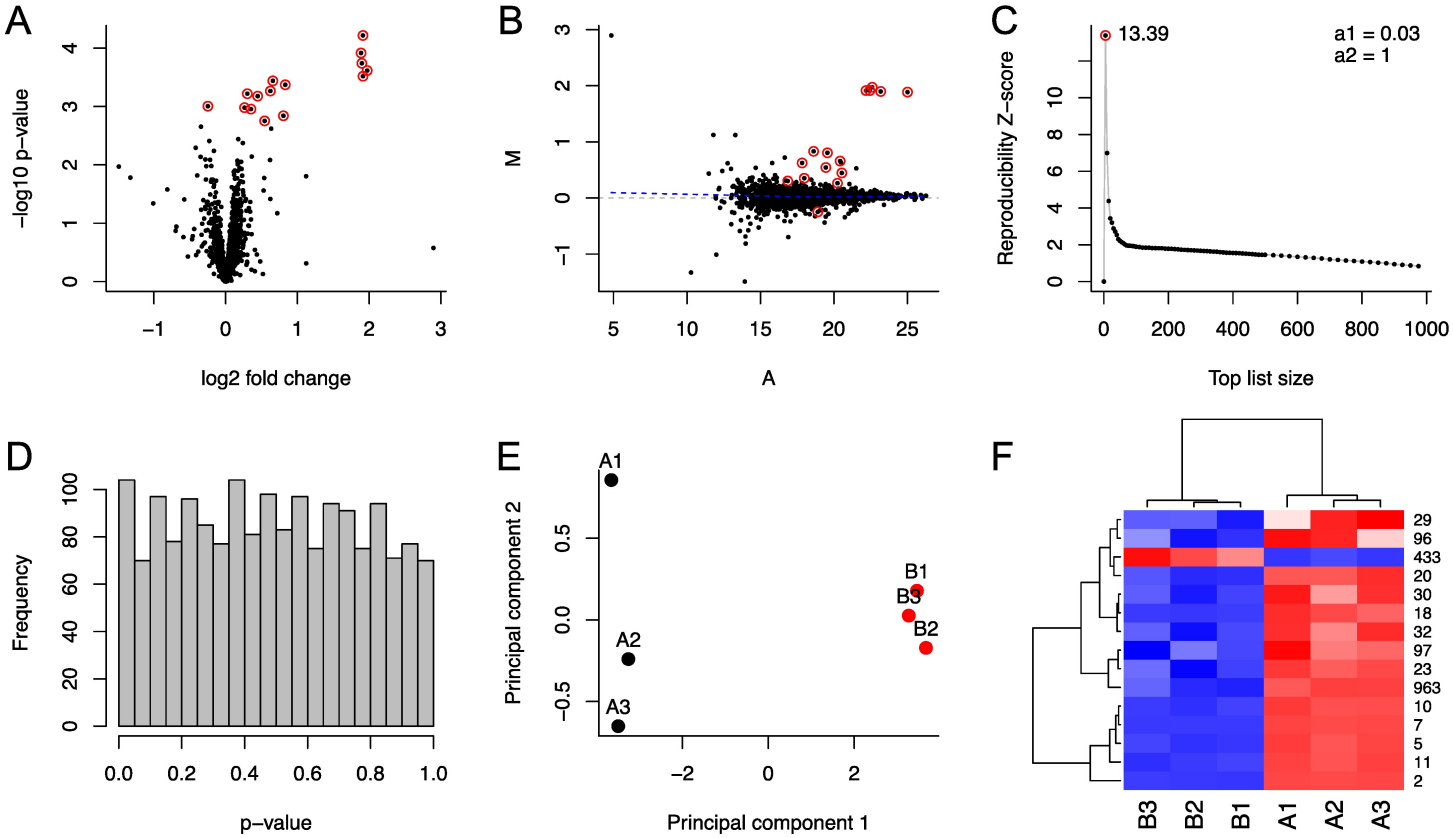
Visualizations provided by ROTS. (A) Volcano plot of the features, where the differentially expressed features are coloured red. (B) MA plot of the features, where the differentially expressed features are coloured red. (C) ROTS reproducibility Z-score as function of top list size. The highest score is marked with red dot together with its value. (D) Histogram of *p*-values. (E) Principal component analysis (PCA) plot of the differentially expressed features. (F) Heatmap and hierarchical clustering of the samples (columns) and the differentially expressed features (rows) using euclidean distance and the complete-linkage agglomerative clustering method.

The ROTS package includes also versatile built-in options for visualization that can be accessed using the standard R plot function. The type of plot can be selected using the type parameter of the function. Fig 1A shows an example of a volcano plot (type=‘volcano’), which visualizes the relationship between fold changes and *p*-values (*i.e.* magnitude of change and statistical significance). It can be used to select the most promising candidate features for further validation studies. Fig 1B shows an example of an MA plot (type=‘ma’), which shows the relationship between the average intensities (A) and intensity ratios (M) calculated across and between the sample groups for each feature, respectively. It can be used, for instance, to assess the quality of normalization used in preprocessing the data. Fig 1C illustrates the ROTS reproducibility Z-score as a function of top list size *k* (type=‘reproducibility’). It can be used to look for possible alternative peaks of Z-score, which could suggest, for example, subgroups of differentially expressed features or artifacts from data normalization. Fig 1D illustrates a histogram of *p*-values (type=‘pvalue’), which enables assessing the overall performance of the hypothesis testing. Under the null hypothesis, *p*-values are uniformly distributed, but if there is a large number of differentially expressed features present, the distribution of *p*-values is likely skewed towards smaller values. Fig 1E shows an example of a principal component analysis (PCA) of the differentially expressed features defined based on a user-specified FDR cutoff (type=‘pca’). It is a transformation, where the data is projected into a new coordinate system of principal components retaining the highest variance. It can be used as a tool to evaluate similarities between samples or groups. By setting the FDR parameter to 1, the principal components are calculated using all the features. Fig 1F illustrates a heatmap with hierarchical clustering of samples and features to visualize the expression levels of differentially expressed features as colours (type=‘heatmap’).

For additional details and examples of using the ROTS package, the reader is referred to the package manual and the three case studies discussed below.

## Results

The benefits of ROTS over other state-of-the-art tools have already been shown in various applications [9–12]. Here, we used three new case studies to further demonstrate the performance of the ROTS method in different study settings, including label-free quantitative proteomics and both bulk and single-cell RNA-seq studies.

### Case study 1: Quantitative label-free proteomics

The ROTS method has previously been benchmarked in label-free shotgun proteomics using spike-in mixtures and complex mouse liver samples, where it has shown competitive performance against other state-of-the-art methods [11]. Here, the performance of ROTS with quantitative mass spectrometry based proteomics data is illustrated in another published benchmark spike-in study, where the truly differentially expressed proteins are known.

The data are from an inter-laboratory spike-in study of the Clinical Proteomic Tumor Analysis Consortium (CPTAC technology assessment study 6) [14–16]. It contains a mixture of 48 human proteins (Sigma UPS1) spiked into a yeast proteome (*S. cerevisiae*) background at different concentration levels ranging from 0.25 to 20 fmol/*μ*L to create five distinct sample groups each with three technical replicates. From the different datasets available, we processed Orbitrap raw files produced at site 86, from which a total of 736 proteins were quantified using the Progenesis software with peptide identifications from the Mascot search algorithm in Proteome Discoverer software. Threshold for peptide identifications was set to FDR < 0.01 and relative protein quantitation was done using non-conflicting peptides, followed by median normalization. Progenesis was unable to align one of the three replicates in one of the sample groups (sample group E). Only the sample groups with all three replicates (sample groups from A to D) were used here for performance benchmarking.

**Fig 2:**
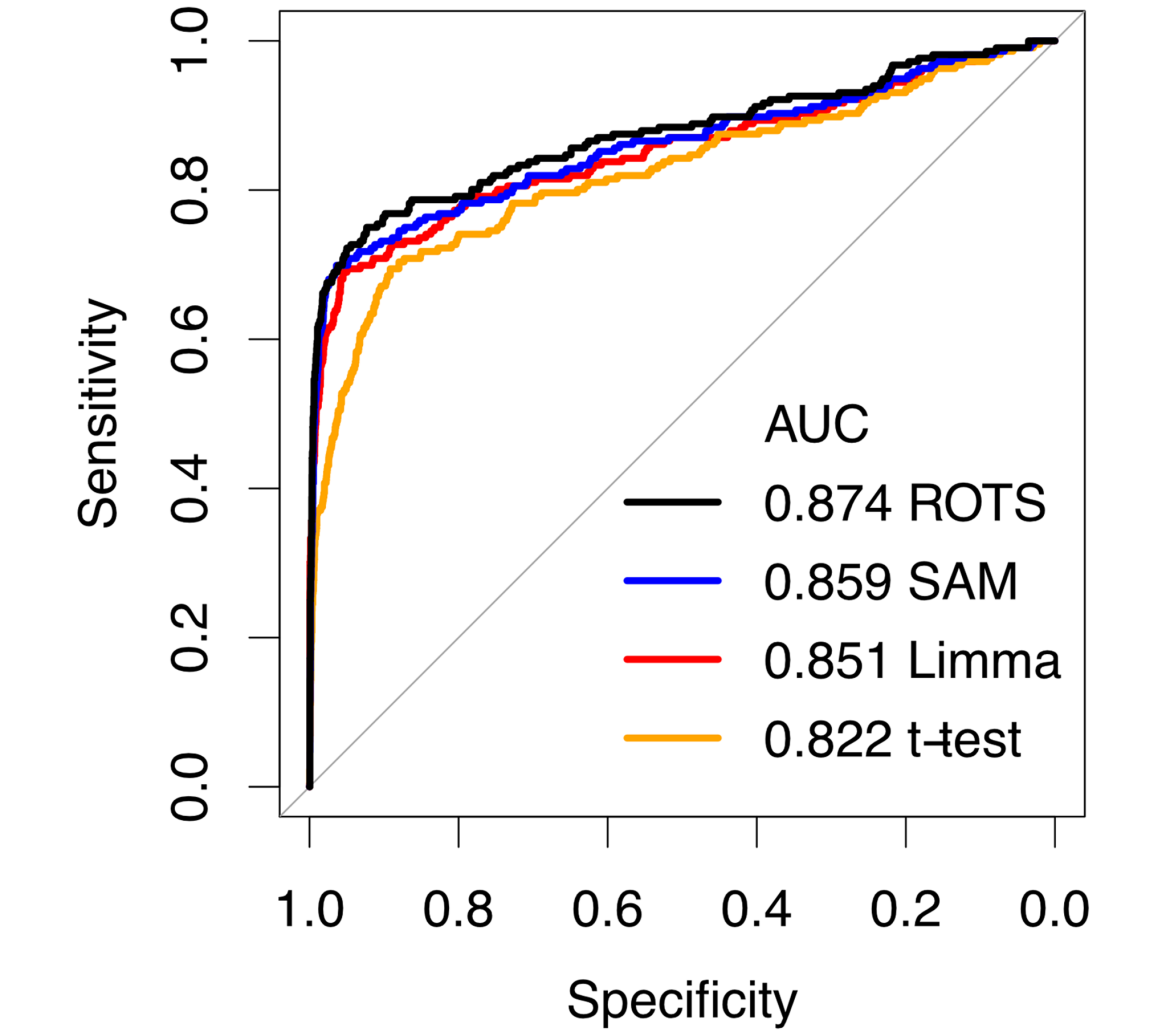
Performance of ROTS and current state-of-the-art methods for proteomics in the spike-in proteomics data from the Clinical Proteomic Tumor Analysis Consortium (CPTAC technology assessment study 6). Performance was evaluated using receiver operating characteristic (ROC) curves and the areas under the curves (AUC).

Fig 2 shows the performance of ROTS on the CPTAC data together with other popular methods for differential expression analysis, including significance analysis of microarrays (SAM) [3], Limma [5] and the Student’s *t*-test. Performance was measured using receiver operating characteristic (ROC) curves, which were created by merging the results from the six possible individual pairwise comparisons involving sample groups from A to D. Overall, ROTS produced a significantly better ROC-curve compared to all other tested methods (DeLong’s test *p* < 0.001 for each method), which supports the applicability of ROTS in proteomics studies to distinguish differentially expressed proteins.

### Case study 2: Bulk RNA-seq

Similarly as with proteomics data, the ROTS method has been extensively benchmarked against other software packages in bulk RNA-seq data [12]. Besides systematically outperforming other methods in spike-in data, ROTS has also been used to successfully identify prognostic markers for clear cell renal cell carcinoma, which confirms the clinical relevance of estimating differential gene expression accurately with ROTS [12]. Here, the performance of ROTS in bulk RNA-seq data is illustrated using a published benchmark spike-in study.

The data are from the sequencing quality control (SEQC) project [17], which includes four distinct sample groups (A, B, C and D) each with five technical replicates sequenced using Illumina HiSeq 2000 platform. For groups A and B, 92 synthetic polyadenylated transcripts provided by the External RNA Control Consortium (ERCC) [18] have been spiked into the Universal Human Reference RNA (UHRR) and Human Brain Reference RNA (HBRR) respectively, so that their concentrations were controlled to have different fold changes of 0.5, 0.67, 1 or 4 between the groups A and B. Samples C and D were then obtained by mixing samples A and B using different ratios: 75% of sample A and 25% of sample B for sample C and vice versa for sample D. For performance benchmarking, we downloaded the count table from GEO with accession number GSE47774. The trimmed mean of M-values (TMM) normalization [20] with voom transformation [19] was applied before differential expression analysis. In total, four comparisons were considered: A vs B, A vs D, B vs C, and C vs D.

**Fig 3:**
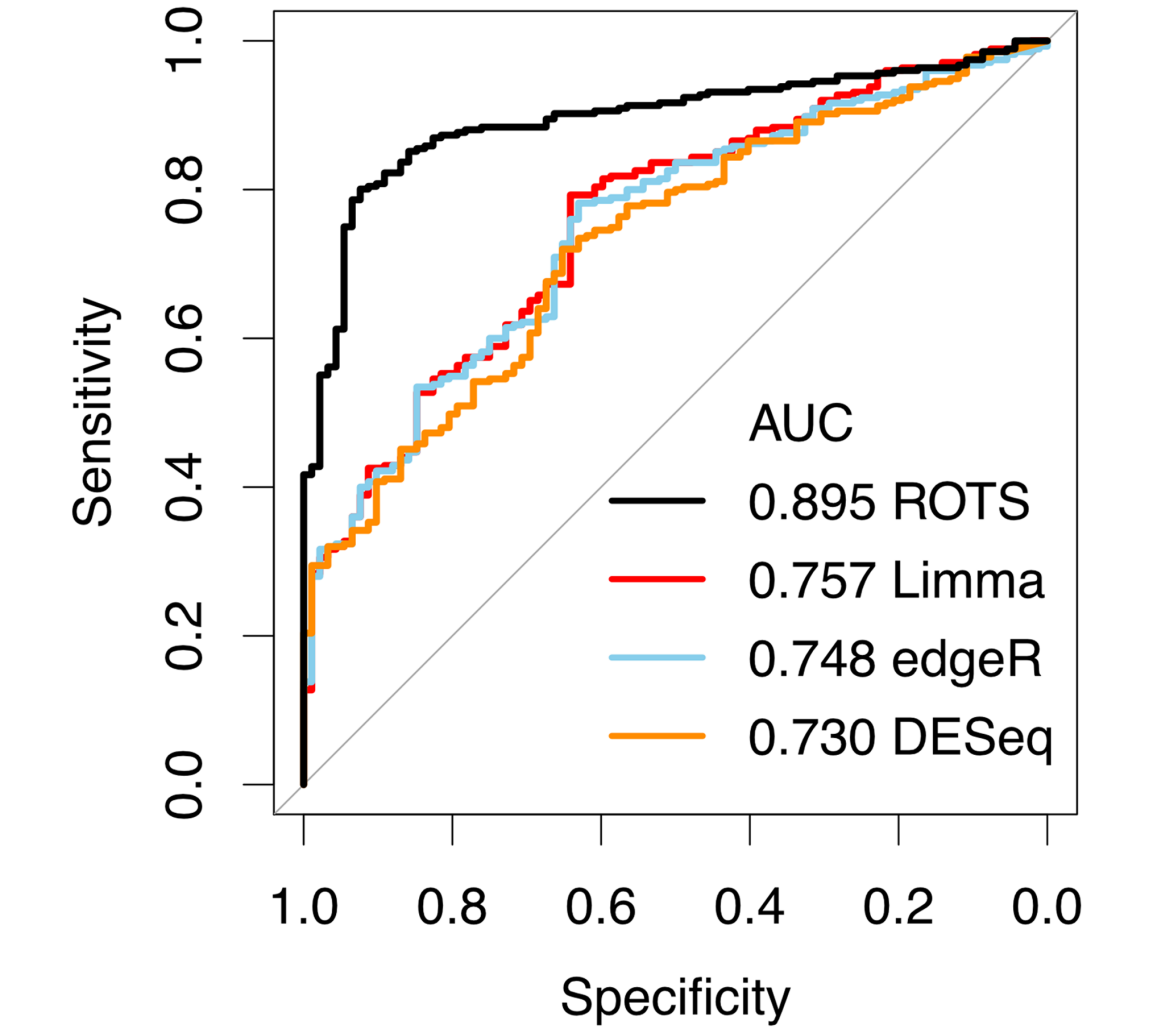
Performance of ROTS and current state-of-the-art methods for bulk RNA-seq in the spike-in data from the SEQC project. Performance was evaluated using receiver operating characteristic (ROC) curves and the areas under the curves (AUC).

Fig 3 shows the performance of ROTS in the bulk RNA-seq data together with other state-of-the-art methods, including edgeR [21, 22], Differential Expression analysis for Sequence count data (DESeq) [23] and Limma [5]. Similarly as with proteomics, the performance was measured using ROC-curves, which were created by merging the results from the individual pairwise comparisons. Again, ROTS showed improved performance over the other tested methods (DeLong’s test *p* < 0.001 for each method), confirming the applicability of ROTS in bulk RNA-seq studies to detect differentially expressed genes.

### Case study 3: Single-cell RNA-seq

Recently, performance of the ROTS method in comparison to other state-of-the-art methods has also been tested in single-cell RNA-seq data. ROTS showed good performance without requiring any single-cell-specific modifications, whereas no systematic benefits of the recent single-cell-specific methods were found [9]. Here, we further demonstrate the utility of the ROTS method also in the increasingly popular single-cell RNA-seq data.

The data are from a previously published single-cell study on innate lymphoid cells (ILC), containing single cell samples sequenced using Illumina HiSeq 2000 platform [24]. Similarly as in our recent study [9], we compared different cell populations. The count table was downloaded from GEO with accession number GSE70580. However, unlike in our previous study, where we compared ILC1 and ILC2 cells against ILC3 cells, the comparison here was performed between ILC1 and ILC2 cells. After excluding cells with total expression < 10000, the data contained 127 ILC1 cells and 139 ILC2 cells. With ROTS we performed TMM normalization and with the other tested methods the guidelines of their respective manuals were followed.

**Fig 4:**
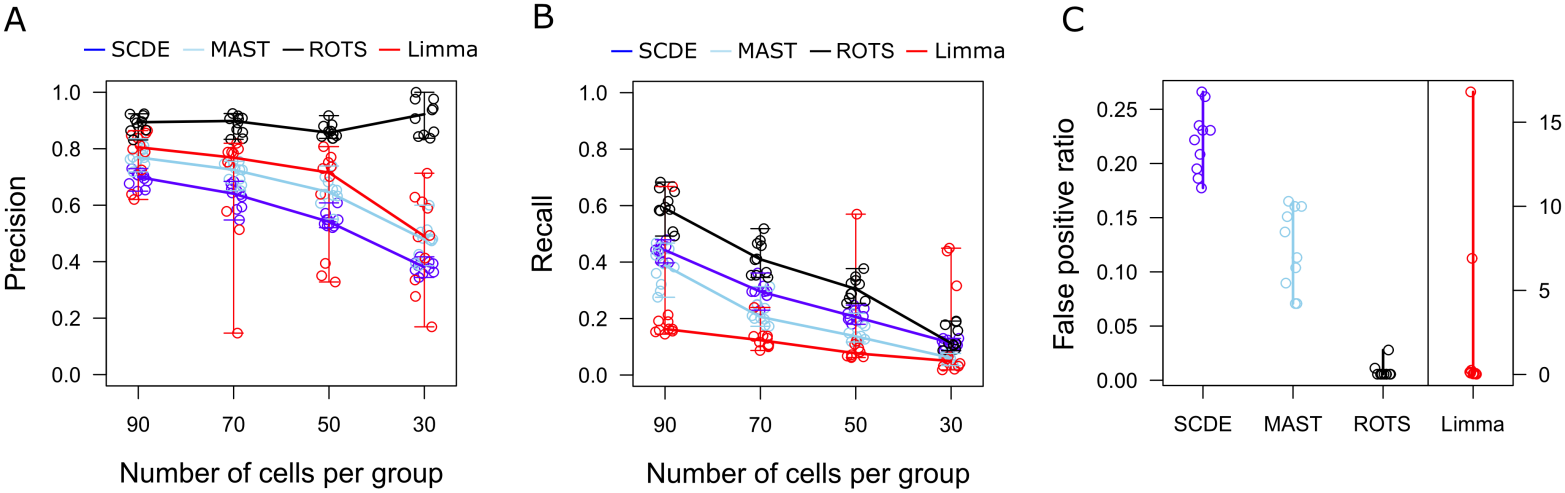
Precision, recall, and false positive ratios of ROTS and current state-of-the-art methods for single-cell RNA-seq in the innate lymphoid cell data. (A) Precision of the findings in reduced data. Precision was defined as the ratio between the number of common detections in the reduced and full data, and the total number of detections in the reduced data. Median values over ten randomly generated subsets are indicated by lines across the different numbers of cells per group. (B) Recall of the findings in reduced data. Recall was defined as the ratio between the number of common detections in the reduced and full data, and the total number of detections in the full data. Median values over ten randomly generated subsets are indicated by lines across the different numbers of cells per group. (C) False positive ratios of the findings in ten randomly generated mock datasets. The false positive ratio was defined as the ratio between the number of differentially expressed genes in the mock comparison and the average number of differentially expressed genes in the actual comparison. Limma was visualized separately because of the different scale compared to the other methods and jittering was used to separate overlapping points.

Fig 4 shows the performance of ROTS in the single-cell RNA-seq data together with other state-of-the art tools, including Single Cell Differential Expression (SCDE) [25], Model-based Analysis of Single-cell Transcriptomics (MAST) [26] and Limma [5]. First, we investigated the precision and recall of the findings when the number of cells was reduced to 90, 70, 50 or 30 cells in both groups. Ten subsets of each size were generated. Overall, ROTS showed the highest precision in finding the genes detected in the full data as differentially expressed (FDR < 0.05) also in the reduced datasets (Fig 4A). Notably, it also had the highest recall, indicating that the findings from the reduced data covered the findings from the full data better than with the other tested methods (Fig 4B). Finally, to investigate whether the methods tended to find a large number of false positives, we generated artificial mock datasets by randomly dividing the 139 ILC2 cells into two groups of similar size ten times. These artificial sets should not differ from one another since all the cells are from the same population. Also the investigation of the mock comparisons ranked ROTS as the top performing method (Fig 4C). These results further confirm the applicability of ROTS for single-cell RNA-seq studies.

## Availability and future directions

ROTS has been successfully applied in multiple studies in a diversity of applications and the results on different types of omics data have shown its overall robustness. A major benefit of ROTS is its ability to automatically select an appropriate test statistic for a specific data under study by maximizing the reproducibility of the differentially expressed features. Therefore, it would be beneficial to integrate ROTS into various existing workflows to perform the differential expression analysis. Besides being able to select a test statistic, ROTS could possibly aid also in selecting, for instance, an appropriate normalization method based on the data. While ROTS is based on a modified *t*-statistic, it is possible to further extend the method by allowing multiple sample groups by using, for example, a modified *F*-statistic. Finally, to enhance the running time of the algorithm, parallelization within the package or improved heuristics could be implemented for optimizing the parameters.

The R package ROTS is freely available from Bioconductor (https://www.bioconductor.org/packages/ROTS) and it conveniently allows to perform statistical testing and result visualization using simple commands. A complete reference manual for the package and a vignette with examples are also available from Bioconductor.
